# Prevalence and Associated Factors of Falls among Older Adults between Urban and Rural Areas of Shantou City, China

**DOI:** 10.3390/ijerph18137050

**Published:** 2021-07-01

**Authors:** Xiaodong Chen, Zeting Lin, Ran Gao, Yijian Yang, Liping Li

**Affiliations:** 1 Injury Prevention Research Center, Shantou University Medical College, Shantou 515041, China; 18xdchen@stu.edu.cn (X.C.); 18ztlin@stu.edu.cn (Z.L.); gaorann@163.com (R.G.); 2 School of Public Health, Shantou University, Shantou 515041, China; 3 Department of Sports Science and Physical Education, Chinese University of Hong Kong, Hong Kong, China; yyang@cuhk.edu.hk; 4 Jockey Club Institute of Ageing, Chinese University of Hong Kong, Hong Kong, China

**Keywords:** older adults, urban and rural areas, falls, associated factors

## Abstract

Background: To investigate the prevalence of falls and associated factors among older adults in urban and rural areas and to facilitate the design of fall prevention interventions. Methods: We used cluster random sampling to investigate the sociodemographic information, living habits, medical status, falls, home environment, and balance ability among 649 older adult participants. Univariate and multivariate logistic regression were used to examine the associated factors of falls. Results: The incidence of falls among older adults in Shantou City was 20.65%. Among them, the incidence was 27.27% in urban areas and 16.99% in rural areas. The rate of injury from falls among older adults was 14.48%, with18.61% in urban area and 12.20% in rural area. Multivariate analysis showed that the associated factors of falls among older adults in Shantou City included a high school or below education level (*OR* = 2.387, 95% *CI*: 1.305–4.366); non-farming as the previous occupation (*OR* = 2.574, 95% *CI*: 1.613–4.109); incontinence(*OR* = 2.881, 95% *CI*: 1.517–5.470); lack of fall prevention education (*OR* = 1.856, 95% *CI*: 1.041–3.311); and reduced balance ability (*OR* = 3.917, 95% *CI*: 2.532–6.058). Discussion: Older adults have a higher rate of falling in Shantou City, compared to the average rate in China. There are similarities and differences in the associated factors of falls among older adults between urban and rural areas of Shantou City. Targeted interventions for older adults in different regions may be more effective in reducing the risk of falls.

## 1. Introduction

According to a report from the World Health Organization (WHO), 646,000 people die from falls every year worldwide, the majority of whom are adults over 65 years old; falls have become the second-largest cause of unintentional injury-related death after road traffic injuries [1]. The WHO notes that falls are the main threat to the health of older adults throughout the world and the leading cause of injuries and injury-related mortality. The consequences of falls among older adults are not only physical trauma and disability but also a series of psychological problems, such as fear of falls, depression and anxiety [2]. The high incidence of falls, disability and mortality in older adults suggests the urgent need for preventative measures.

Falling is the leading cause of injury-related death among older adults in China [3]. According to China’s National Bureau of Statistics, by the end of 2018, approximately 249 million Chinese citizens were older adults aged 60 and over, accounting for 17.9% of the population; among these, approximately 167 million individuals were aged 65 and over, accounting for 11.9% of the total population [4]. Based on China’s national statistics, about 40 million older adults fall at least once a year in China. The direct medical cost of falls among older adults in China is more than 5 billion yuan per year and will continue to increase with the increasing aging population [5].

Falls among older adults are the result of the interaction of various factors. According to previous studies, these consist of both internal and external risk factors. Internal risk factors include physiological factors (e.g., decreased balance ability and vision), pathological factors (e.g., stroke, Parkinson’s disease), pharmaceutical factors (e.g., antidepressant use, hypoglycemic agent use), and psychological factors (e.g., depression, anxiety). External factors include environmental factors (e.g., dim lighting, lack of handrails in bathrooms) and social factors (e.g., low income, living alone) [6]. To date, there are many studies on falls among older adults in Europe, Australia and other developed countries, and some interventions have been carried out to reduce the occurrence of falls and fall-related injuries among older adults [7,8,9]. However, there is a lack of studies considering the influence of external environment on the falls among older adults in China [6]. Moreover, due to the differences in culture and socioeconomic status, the associated factors of falls among older adults in China may be different from those in Europe, Australia and other developed countries, which suggests a need for further investigation.

According to the sixth census in China (2010), the size of the rural population is 660 million, among whom nearly 100 million individuals are over 60 years old, accounting for 15.15% of the rural population [10]. Existing domestic research has mainly focused on the investigation of older adults in developed cities, including Beijing, Shanghai, and Shenzhen [11,12,13]. There is insufficient investigation on falls of older adults in rural areas. Older adults in rural areas are an underserved group, as many policies and services for older adults are prioritized for those living in urban areas, and medical conditions, income levels and living environments in rural areas are considered worse than their urban counterparts. At the same time, the cultural levels, living habits and environments of older adults in urban and rural areas are also different [14,15], which may cause them to fall differently. Therefore, the aim of this study was to investigate the fall incidence among older adults in Shantou City and to compare the different associated factors of falls between urban and rural areas so as to guide fall prevention and health decision-making for these two populations of older adults.

## 2. Methods

### 2.1. Subjects

Older adults were recruited from the urban and rural areas of Shantou City, China. Shantou is a medium-size city with both urban and rural areas. The socioeconomic status, environments and mobility pattern of older adults in urban and rural areas of Shantou are similar to those in many other regions of China. Our inclusion criteria of participants were (1) age ≥ 60 years; (2) ability to walk independently or rely on auxiliary facilities; (3) residence in the local area for 1 year; (4) ability to understand the survey and cooperate with the completion of the assessment; and (5) willingness to participate in the survey and research and ability to sign informed consent. Exclusion criteria included older adults who (1) were unable to walk in the past year; (2) had severe illness or dysfunction; (3) were unable to complete medical examinations and assessment projects; and (4) had cognitive dysfunction and thus an inability to answer questions correctly.

Definition of fall

A fall is a sudden involuntary or unintentional change of body position, resulting in a drop to the ground or a lower plane [6]. According to the classification of falls from the International Classification of Diseases (ICD-10), falls include the following two categories: (1) a fall from one plane to another and (2) a fall onto the same plane [6].

### 2.2. Study Design

**Figure 1:**
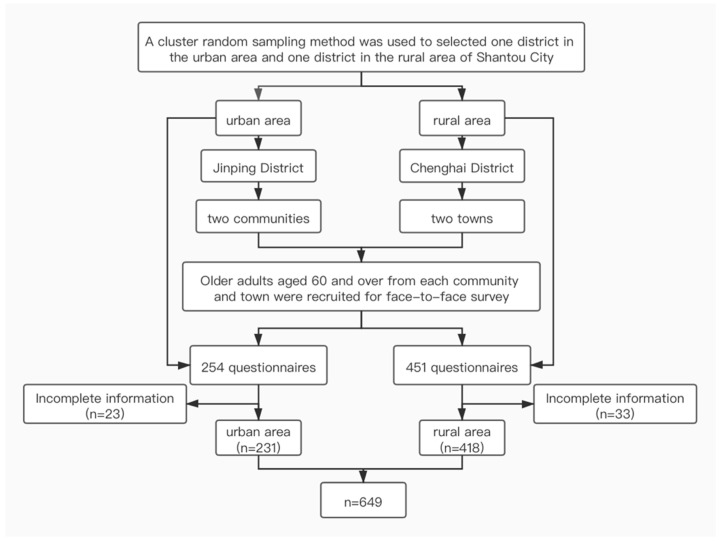
Flowchart of this study.

We conducted a cross-sectional study from April 2018 to April 2019. A cluster random sampling method was used to select one district in the urban area and one district in the rural area of Shantou City, and then select two communities (urban area) and two towns (rural area) respectively (Figure 1). Older adults aged 60 and over from each community and town were recruited for face-to-face survey/interview. In addition, we evaluated the home environment of the older adults and tested their balance ability (see details in Section 2.3). The researchers filled out the questionnaires because older adults with low education levels and poor visual acuity could not fully understand the content of the questionnaire by themselves. Interviews were conducted in the participants’ homes by trained researchers using local dialects in plain language. Since the proportion of older adults in rural areas in Shantou City is larger than of older adults in urban areas, more questionnaires were distributed to the rural areas. A total of 705 (urban area = 254, rural area = 451) questionnaires were issued and 649 (urban area = 231, rural area = 418) were completed (Figure 1).

### 2.3. Data Collection

Demographic information included sex, age, occupation, and education level of the participants. Health status included hearing, vision, medication, and incontinence. Information on fall situations included the incidence of falls and injuries related to falls within one year, which was obtained from a self-report form. If the participant had fallen within the past year, a fall situation form was required to be completed. The fall situation form included fall times, locations, and severity, etc. Information about living environment was based on a survey from the “Evaluation Form for Preventing Older Adults from Falling in the Home Environment Risk Factors” [6] via direct observation by the researcher, including (1) the floors and passageways (whether sundries are piled up in the corridor, whether antiskid floor tiles are used, whether pets are kept, etc.); (2) the living room (whether the lighting is sufficient, whether the commonly used chairs have armrests, whether the height and hardness of the sofa are suitable for getting up, etc.); (3) the bedroom (whether there is a double control lighting switch, whether there is a telephone installed at the head of the bed, whether there are sundries beside the bed that affect getting on and off the bed, etc.); (4) the kitchen (whether it is well ventilated and has a telephone installed, etc.) and (5) the bathroom (whether the drainage is smooth, whether there are antiskid pads, whether there are handrails beside the toilet, etc.). The above five parts were scored respectively (1 = not up to standard, 0 = up to standard, and the total score ranges from 0–5 points). The number of home environment issues in our study was categorized into three levels: (1) ≤1: at most one part of the home is not up to standard; (2) 2–3: two or three parts are not up to standard; and (3) >3: more than three parts are not up to standard. The higher the total score, the greater the risk of the home environment. We also used the Older Adults Balance Ability Test Scale to assess the balance ability of older adults and their risk of falling [6]. Measurements were selected from the “Older Adults Balance Ability Test Scale” in the “Technical Guide for Falling Interventions for Older Adults”, which include the following three parts: (1) static balance ability; (2) posture control ability; and (3) dynamic balance ability. The classification of the scale was as follows [6]: (1) 17–20 points: good balance ability; (2) 13–16 points: acceptable balance ability but increased risk of falling; (3) 7–12 points: greatly weakened balance ability and greater risk of falling; and (4) 0–6 points: poor balance ability and greatest risk of falling and injury. The balance ability in our study was categorized into two levels: (1) normal balance ability: 17–20 points, (2) reduced balance ability: 0–16 points.

### 2.4. Ethics

This study was approved by the Ethics Committee of Shantou University Medical College (No. SUMC-2018-41). All participants gave their informed consent and volunteered to participate.

### 2.5. Data Analysis

EpiData 3.1 was used for double data entry verification. SPSS 23.0 software (SPSS Inc., Chicago, Illinois, USA) was used for data analysis. Classification (categorical) data are described by frequency and percentage. Measurement (continuous) data are described by the mean and standard deviation (X ± SD). Chi-square test was used for univariate analysis to examine the effect of each explanatory variable on the probability of falls. Significant explanatory variables (*p* < 0.05) from the univariate analysis as well as age and sex were included in the multivariate logistic regression analysis in order to identify the associated factors of falls. Results from the final model included odds ratio (*OR)* and its 95% confidence interval (*CI).* A two-tailed *p*-value of less than 0.05 was considered statistically significant. 

## 3. Results

A total of 649 older adults were surveyed from urban and rural areas in this study. A total of 705 questionnaires were originally issued and 649 were finally completed, resulting in an effective rate of 92.06%. The effective rates in urban and rural areas were 90.94% (231/254) and 92.68% (418/451), respectively. The survey respondents in the urban areas were 60–83 years old, with an average age of 67.39 (SD 4.61) years, and the survey respondents in the rural areas were 60–88 years old, with an average age of 72.71 (SD 7.62) years. 

### 3.1. The Incidence of Falls and Injury Rates among Older Adults in Shantou City

Among the 649 older adult participants, 134 had fallen at least once in the past year, an incidence of 20.65%. Among them, the incidence was 27.27% in urban areas and 16.99% in rural areas. The rate of injury from falls among older adults was 14.48%, with18.61% in urban area and 12.20% in rural area. Table 1 shows the falls and injury rates in both urban and rural areas.

### 3.2. Analysis of Associated Factors of Falls among Older Adults in the Past Year

#### 3.2.1. Univariate Analysis of Associated Factors of Falls among Older Adults in Shantou City

Univariate analysis showed that ten factors were associated with falls among older adults in Shantou City in the past year, including area (urban or rural), education level, previous occupation, whether they had taken calcium or vitamin D, whether they had incontinence, whether they used canes, balance ability, whether they were afraid of falling, belief on whether fall can be prevented, and whether they had received fall prevention education (*p* < 0.05). Six factors were associated with falls among older adults in urban areas, including education level, whether they took diabetes drugs, whether they had incontinence, balance ability, whether they were afraid of falling, and whether they had received fall prevention education (*p* < 0.05). Six factors associated with falls among older adults in rural areas included their previous occupation, whether they had incontinence, whether they used canes, living environment issues, balance ability, and whether they were afraid of falling (*p* < 0.05) (see Table 2).

#### 3.2.2. Multivariate Analysis of Associated Factors of Falls among Older Adults in the Past Year

Significant explanatory variables (*p* < 0.05) from the univariate analysis as well as age and sex were included in the multivariate logistic regression analysis. The dependent (outcome) variable was whether or not a fall occurred. The explanatory variable assignment is shown in Table 3. Multivariate logistic regression analysis showed that low education level, non-farming as the previous occupation, incontinence, lack of fall prevention education, and poor balance ability were associated with risk of falls among the older adults in Shantou City. Low education level, diabetes medication use, incontinence, lack of fall prevention education, and poor balance ability were associated with risk of falls among the urban older adults. Non-farming as the previous occupation, incontinence, home environment issues, and poor balance ability were related to the risk of falls among the rural older adults (Table 4).

## 4. Discussion

The incidence of falls among older adults in Shantou City in the past year was 20.65%, which was higher than the result of a meta-analysis in China (18.3%) [16], but lower than that of Cape Town (26.4%) in South Africa [17] and close to that of South Korea (20.95%) [18] and Japan (20.8%) [19]. The fall rate and rate of injury from falls among older adults in urban areas were different from those among older adults in rural areas. This is consistent with studies by Li [13] and He [15]. A possible reason is that the social demographic factors of older adults in urban and rural areas of Shantou City are different. For example, the education level of older adults in urban areas with a college or above degree accounted for 25.54%, but only 15.07% in rural areas. At the same time, 55.0% of the older adults in rural areas are farmers, and long-term physical exercise of farmers may have a protective effect on falls [20]. On the other hand, only 3.5% of the older adults in urban areas are farmers. Our results suggest the need to carry out intervention measures according to specific fall circumstances in different regions.

Incontinence was a risk factor for falls in both urban (*OR* = 4.717) and rural areas (*OR* = 2.462). This is consistent with the results from Choi and Kim’s studies [18,21], indicating that incontinence is a common risk factor of falls across different regions and populations. Incontinence causes a corresponding increase in the frequency of urination, which is increased further due to an increased water intake resulting from the hot climate and the rich tea culture in Shantou. The frequent urination, especially at night, greatly increases the risk of falling. Therefore, targeted intervention for older adults with incontinence or urinary system infection may effectively prevent and reduce the risk of falls.

Decreased balance ability was a risk factor of falls in both urban (*OR* = 3.573) and rural areas (*OR* = 4.250) of Shantou City. Our finding is consistent with the results of Zhao and Hou’s studies [22,23], in which they found that the balance ability of older adults decreases with age. This is due to the structural and functional impairment and degradation of the bones, joints, ligaments and muscles with age [6]. At the same time, degeneration of the central nervous system and osteoporosis affect older adults’ muscle strength, sensation, coordination, reaction times, and ability to move [6]. All of these factors contribute to an increased risk of falling. Physical exercise has been shown to improve balance ability in older adults, including Taijiquan [24]. Regular physical exercise helps enhance muscle strength and balance ability [20]. In addition, group-based exercise helps older adults interact and communicate with peers, which improves their psychosocial health and confidence, and thus reduces their fear of falling [25].

Older adults with lower education levels and those who lack fall prevention education have a higher risk of falling. The *OR*s for these two factors were 2.387 and 1.856 for the urban area, respectively. This is consistent with the findings of Huang [26]. The proportion of older adults who have received fall prevention education in Shantou City is only 16.80%. Older adults with higher education levels generally have a higher standard of living and a greater sense of self-protection [27]. Older adults with low education levels are less aware of the knowledge of fall prevention, which may increase their exposure to falls. For older adults in rural areas, due to their overall low level of education and limited access to fall prevention education, the level of education and whether they had received fall prevention education were not significantly associated with the incidence of falls. Our results suggest that more education programs should be provided to older adults to prevent falls, especially in less educated and underdeveloped areas. More attention should be paid to the popularization and dissemination of fall prevention programs, and targeted health education should be carried out to improve the awareness among older adults to prevent falls.

Taking diabetes medication increased the risk of falls by 3.842 times compared to not taking diabetes medicine, which is consistent with the results of Berlie’s study [28]. Falls occur in older adults after taking too much diabetes medication. Studies have found that patients with diabetes have twice the risk of falls than those without diabetes [29]. Older adults with diabetes often take multiple drugs for many years. One study showed that the side effects of diabetes drugs (e.g., dizziness, anxiety) can increase the risk of falls in older adults [30]. Older adults have weakened memory, take multiple chronic medications daily, and lack correct guidance from a doctor. In the process of taking the medication, they are prone to inappropriate doses that may lead to an increased risk of falls. Therefore, caregivers should pay attention to the daily medication of older adults and provide guidance and education on medication management, especially those with diabetes.

Previous occupation as a farmer was a protective factor for falls among rural older adults. One explanation is that past engagement in farming was a form of physical exercise, which may have improved their physical fitness over time [13]. However, when we compared and analyzed previous occupation and balance ability, we did not find an association between older adults who used to be farmers and their balance ability. The possible reason is that the cause of falling is affected by many factors (e.g., strength, reaction time) and does not only depend on the level of balance ability. On the other hand, few older adults in urban areas were previously employed as farmers, which may explain the lack of statistical significance of their occupation on fall risk.

This study found that rural older adults who had more than three home environment issues had a 3.459-fold higher risk of falls than those with one or no home environment issue. Sophonratanapokin’s study [31] also indicated that a high number of home environment issues was a risk factor for falls among older adults. Our investigation found that the living environment of rural older adults in Shantou City is much worse than that of urban older adults. The living environment of rural older adults (floor and passageways, living room, bedroom, kitchen, and bathroom) is littered with many potential safety hazards. Some households even lack separate toilets, which greatly increases the risk of falls among older adults. In addition, this study found that 28.2% of rural older adults lived alone, and nearly half of falls occurred at home. Therefore, there is an urgent need to conduct a comprehensive assessment of the home environment of older adults and identify hazards to reduce their risk of falls.

The limitations of this study mainly include the following: First, the questionnaire survey covered falls in the past year, which could have been affected by the declined memory of older adults and therefore recall bias. However, this is a common issue for most survey studies. Considering that older adults in Shantou City are not well educated, especially rural older adults who are mostly illiterate, the questionnaires were filled out by the researchers who asked the questions to the older adults. Our investigators have been trained for consistency in data collection to reduce subjectivity. To address the memory problems of older adults, the researchers made multiple inquiries in local dialects to reduce recall bias. Second, falls among older adults are the result of the interaction of multiple factors. This study mainly focused on investigating the internal risk factors and home environment of older adults. Future research may evaluate the psychological factors and comprehensive social factors among older adults in urban and rural areas.

## 5. Conclusions

Older adults have a higher rate of falling in Shantou City, compared to the average rate in China. Our focus for fall prevention interventions should target older adults with low education level, non-farming as the previous occupation (especially in rural areas), incontinence, lack of fall prevention education, and poor balance ability. There are similarities and differences in the associated factors of falls among older adults between urban and rural areas of Shantou City. For instance, a lack of fall education and diabetes medication are risk factors for falls in urban older adults, while home environment issues are a risk factor for falls among rural older adults. Our results suggest that targeted interventions for different older adult populations may be more effective in reducing the risk of falls. 

## Figures and Tables

**Table 1:** The incidence of falls and injury rates among older adults in Shantou City.

Area	Fall (*n*)	Fall Rate (%)	Injuries (*n*)	Injury Rate (%)
All	134	20.65	94	14.48
Urban areas	63	27.27	43	18.61
Rural areas	71	16.99	51	12.20

**Table 2:** Univariate analysis of associated factors of falls among older adults in Shantou City in the past year.

Variable	All (*n* = 649)	χ^2^	*p*-Value	Urban Areas (*n* = 231)	χ^2^	*p*-Value	Rural Areas (*n* = 418)	χ^2^	*p*-Value
	Fall			Fall			Fall		
Areas		9.610	0.002 *						
Urban	63 (27.27)								
Rural	71 (16.99)								
Sex		3.035	0.082		0.547	0.459		2.445	0.118
Male	33 (16.50)			16(23.88)			17(12.78)		
Female	101(22.49)			47(28.66)			54(18.95)		
Age		0.816	0.665		0.364	0.834		3.282	0.194
60–69	90(19.91)			48(28.24)			42(14.89)		
70–79	37(23.13)			13(24.07)			24(22.64)		
≥80	7(18.92)			2(28.57)			5(16.67)		
Education level		5.203	0.023 *		7.513	0.006 *		0.967	0.325
High school and below	118(22.39)			55(31.98)			63(17.75)		
College and above	16(13.11)			8(13.56)			8(12.70)		
Previous occupation		10.857	0.001 *			0.687 ^#^		6.021	0.014 *
Farmer	102(24.58)			3(37.50)			29(12.83)		
Non farmer	32(13.68)			60(26.91)			42(21.88)		
Living alone		0.000	0.989		0.393	0.531		1.311	0.252
No	104(20.63)			57(27.94)			47(15.67)		
Yes	30(20.69)			6(22.22)			24(20.34)		
Calcium or Vitamin D		4..205	0.040 *		1.401	0.236			1.000^#^
No	112(19.48)			44(25.29)			68(16.96)		
Yes	22(29.73)			19(33.33)			3(17.65)		
Vision		0.142	0.707		0.003	0.956		1.161	0.281
Normal	56(21.37)			40(27.40)			16(13.79)		
Weaken	78(20.16)			23(27.06)			55(18.21)		
Dizziness			0.158 ^#^			0.349 ^#^			1.000 ^#^
No	131(20.40)			60(26.67)			71(17.03)		
Yes	3(42.86)			3(50.00)			0(0.00)		
Diabetes		0.260	0.610		0.869	0.351		0.383	0.536
No	120(20.94)			58(26.61)			62(17.46)		
Yes	14(18.42)			5(38.46)			9(14.29)		
Hypertension		1.976	0.160		3.388	0.066		1.050	0.306
No	83(19.08)			43(24.29)			40(15.50)		
Yes	51(23.83)			20(37.04)			31(19.38)		
Heart disease		2.493	0.114		0.102	0.749			1.000 ^#^
No	123(22.45)			45(32.85)			78(18.98)		
Yes	30(29.70)			29(30.85)			1(14.29)		
Hypotension		1.428	0.232		1.322	0.250			1.000 ^#^
No	128(20.32)			58(26.48)			70(17.03)		
Yes	6(31.58)			5(41.67)			1(14.29)		
Diabetes medicine		1.018	0.313		6.704	0.023 *		0.000	1.000
No	117(20.10)			55(25.35)			62(16.99)		
Yes	17(25.37)			8(57.14)			9(16.98)		
Hypertension medicine		2.333	0.127		0.230	0.632		1.008	0.315
No	85(19.02)			43(24.16)			42(15.61)		
Yes	49(24.26)			20(27.03)			29(19.46)		
Incontinence		14.238	>0.001 *		5.702	0.017 *		8.126	0.004 *
No	113(22.24)			53(25.12)			60(15.50)		
Yes	21(41.18)			10(50.00)			11(35.48)		
Cane		4.601	0.032 *			1.000 ^#^		8.533	0.014 *
No	117(19.63)			62(27.31)			55(14.9)		
Needed	17(32.08)			1(25.00)			16(30.3)		
Home environment issues		2.087	0.352		5.080	0.079		7.685	0.021
≤1	60(21.66)			37(33.94)			23(13.69) ^a^		
2–3	56(18.54)			22(22.68)			34(16.59) ^a,b^		
>3	18(25.71)			4(16.00)			14(31.11) ^b^		
Balance ability		42.891	<0.001 *		14.65	<0.001 *		26.152	<0.001 *
Normal	35(10.51)			16(15.09)			19(8.37)		
Reduced	99(31.33)			47(37.60)			52(27.23)		
Fear of falling		5.753	0.016 *		5.697	0.017 *		4.432	0.035 *
Not afraid	49(16.50)			30(21.58)			19(12.03)		
Afraid	85(24.15)			33(35.87)			52(20.00)		
Can fall be prevented		6.806	0.033 *		2.959	0.228		0.256	0.880
No	80(18.91) ^a^			26(27.08)			54(16.51)		
Yes	35(20.59) ^a,b^			19(22.35)			16(18.82)		
Do not know	19(33.93) ^b^			18(36.00)			1(16.67)		
Fall prevention education		4.693	0.030 *		4.456	0.035 *		0.682	0.409
No	117(22.32)			59(29.80)			58(17.79)		
Yes	17(13.60)			4(12.12)			13(14.13)		

Notes: * *p* <0.05, ^#^ Fisher’s exact test. ^a,b^: Different letter represents a statistically significant difference in rates between the two groups.

**Table 3:** Variable assignment for the multivariate logistic regression analysis.

Variable	Assignment
Areas	Rural = 1, Urban = 2
Sex	Male = 1, Female = 2
Age	1 = 60–69, 2 = 70–79, 3 = ≥80
Education level Previous occupation	College and above = 1, High school and below = 2 Non-farmer = 1, Farmer = 2
Calcium or Vitamin D	Yes = 1, No = 2
Diabetes medicine	No =1, Yes =2
Incontinence	No =1, Yes =2
Cane	No =1, Yes =2
Home environment issues	1 = ≤1, 2 = 2–3, 3 = >3
Balance ability	Normal = 1, Reduced = 2
Fear of falling	Afraid = 1, Not afraid = 2
Can fall be prevented	Yes =1, Do not know = 2, No =3
Fall prevention education	No = 1, Yes = 2

Notes: Home environment issues: ≤1: at most one part of the home is not up to standard; 2–3: two or three parts are not up to standard; >3: more than three parts are not up to standard.

**Table 4:** Multivariate analysis of associated factors of falls among older adults in the past year.

Factor	*β*	SE	Wald	*p*-Value *	*OR*	95% *CI*
All						
Education level (reference: College and above)	0.870	0.308	7.980	0.005 *	2.387	1.305–4.366
Previous occupation (reference: Farmer)	0.946	0.239	15.715	<0.001 *	2.574	1.613–4.109
Incontinence	1.058	0.327	10.715	0.001 *	2.881	1.517–5.470
Fall prevention education	0.619	0.295	4.389	0.036 *	1.856	1.041–3.311
Balance ability (reference: Normal)	1.365	0.223	37.644	<0.001 *	3.917	2.532–6.058
Urban areas						
Education level (reference: College and above)	1.314	0.458	8.253	0.004 *	3.722	1.518–9.126
Diabetes medicine use	1.346	0.608	4.905	0.027 *	3.842	1.167–12.643
Incontinence	1.551	0.554	7.831	0.005 *	4.717	1.592–13.979
Fall prevention education	1.203	0.596	4.079	0.043 *	3.331	1.036–10.710
Balance ability (reference: Normal)	1.273	0.348	13.386	<0.001 *	3.573	1.806–7.069
Rural areas						
Previous occupation (reference: farmer)	0.885	0.286	9.579	0.002 *	2.422	1.383–4.242
Incontinence (reference: No)	0.901	0.437	4.249	0.039 *	2.462	1.045–5.800
Home environment issues (reference: ≤1)			8.344	0.015 *		
2–3	0.324	0.308	1.103	0.294	1.382	0.756–2.528
>3	1.241	0.430	8.311	0.004 *	3.459	1.488–8.042
Balance ability (reference: Normal)	1.447	0.301	23.091	<0.001 *	4.250	2.355–7.668

Notes: *OR*: odds ratio, 95% *CI*: 95% confidence interval. * *p* < 0.05.

## Data Availability

The datasets used and analyzed in the study are available from the corresponding author on reasonable request.
